# How to increase response rate to a questionnaire study?

**DOI:** 10.4103/0253-7613.75687

**Published:** 2011-02

**Authors:** Jaykaran

**Affiliations:** Department of Pharmacology, Government Medical College, Surat, Gujarat, India. E-mail: drjaykaran@yahoo.co.in

Sir,

I read with interest the article entitled “Attitude and opinion towards essential medicine formulary” by Sharma *et al*.[1] In this study, the authors mentioned that out of 200 doctors who were approached, only 90 (45%) completed the questionnaire. It is also said, “non responders constitute a major problem in such surveys”. However, it appears that in those studies where response rate is low, chances of bias are high as only those subjects who felt strongly about the survey question may have responded positively and therefore the results obtained may not be generalized. Several journals do not publish a paper if the response rate is less than 70%.[2] Several measures that can be adopted to increase the response rate are as follows[3] :

1. Questionnaire should be clearly designed and should have a simple layout.
2. Some incentives or prize should be offered to the participants in return for completion.
3. It should be adequately piloted and tested.
4. Participants should be notified in advance and invitation should be sought.
5. Aims of the study and means of completing the questionnaire should be clearly explained to participants.
6. In case of postal questionnaires, a stamped addressed envelope should be included.
7. Researcher should be present on site to answer queries raised by participants and collect the completed questionnaire.
8. Participants should feel that they are a stakeholder in the study.
9. Questions should be framed in such a way that they attract the participant attention.
10. Questionnaire should be concise and should have clear focus and purpose.
11. Questionnaire should be clear, unambiguous and appealing to look at.

In cases of studies with lower response rate, it would help to compare characteristics of people who responded with the people who did not respond. If there is significant difference of characteristics between the groups, the results cannot be generalized. On the other hand, if there is no significant difference between the groups, the results can be generalized even if the response rate is low.[2]
